# Airflow as a Possible Transmission Route of Middle East Respiratory Syndrome at an Initial Outbreak Hospital in Korea

**DOI:** 10.3390/ijerph15122757

**Published:** 2018-12-06

**Authors:** Minki Sung, Seongmin Jo, Sang-Eun Lee, Moran Ki, Bo Youl Choi, JinKwan Hong

**Affiliations:** 1 Department of Architectural Engineering, Sejong University, 209, Seoul 05006, Korea; mksung@sejong.ac.kr (M.S.); joseongmin01@naver.com (S.J.); 2 Division of Vectors and Parasitic Diseases, Korea Centers for Disease Control and Prevention, Cheongju 28159, Korea; ondalgl@korea.kr; 3 Department of Cancer Control and Population Health, Graduate School of Cancer Science and Policy, National Cancer Center, Goyang 10408, Korea; moranki@ncc.re.kr; 4 Department of Preventive Medicine, Hanyang University Medical College, Seoul 04763, Korea; bychoi@hanyang.ac.kr; 5 Department of HVAC & Firefighting Engineering, Gachon University, Seongnam 13120, Korea

**Keywords:** Middle East Respiratory Syndrome, infection outbreak, airflow analysis, transmission route, ventilation, tracer gas

## Abstract

In this study, the results of an airflow investigation conducted on 7 June 2015 as part of a series of epidemiologic investigations at Pyeongtaek St. Mary’s Hospital, South Korea, were investigated. The study involved 38 individuals who were infected directly and indirectly with Middle East Respiratory Syndrome (MERS), by a super-spreader patient. Tracer gas experiments conducted on the eighth floor, where the initial patient was hospitalized, confirmed that the tracer gas spread to adjacent patient rooms and rooms across corridors. In particular, the experiment with an external wind direction and speed similar to those during the hospitalization of the initial patient revealed that the air change rate was 17–20 air changes per hour (ACH), with air introduced through the window in the room of the infected patient (room 8104). The tracer gas concentration of room 8110, which was the farthest room, was 7.56% of room 8104, indicating that a high concentration of gas has spread from room 8104 to rooms across the corridor. In contrast, the tracer gas was barely detected in a maternity ward to the south of room 8104, where there was no secondary infected patient. Moreover, MERS is known to spread mainly by droplets through close contact, but long-distance dispersion is probable in certain environments, such as that of a super-spreader patient hospitalized in a room without ventilation, hospitals with a central corridor type, and indoor airflow dispersion due to external wind.

## 1. Introduction

A Korean businessman returning from a business trip to the Middle East was admitted to Pyeongtaek St. Mary’s Hospital, South Korea, from 15 to 17 May 2015. He was confirmed to be infected with Middle East Respiratory Syndrome (MERS) on 20 May and spread the infection both directly and indirectly, resulting in 186 infections and 38 deaths in Korea [1]. By 23 December 2015, which was officially declared as the end of the MERS outbreak [2], approximately 17,000 suspected patients were isolated, and Korea suffered significant economic losses estimated at US$8.5 billion [3].

An estimate from the transmission model by Zhang et al. [4] revealed a higher reproductive number for MERS outbreaks in South Korea than other small outbreaks, which was mainly related to healthcare-associated settings. According to Choi [5], approximately 80% of the total MERS patients in South Korea were infected in healthcare facilities by four super-spreaders, who infected more than five individuals each [6]. This indicates the importance of the isolation and treatment of super-spreaders using prompt epidemiologic investigations for the prevention of large-scale infections. Park et al. [6] and Kim et al. [7] analyzed the epidemiologic characteristics of 36 secondary and tertiary patients infected by the initial super-spreader patient at Pyeongtaek St. Mary’s Hospital, and suggested the necessity of additional investigations into the cases of infected patients, for whom close contact was not confirmed as the infection route. Cho et al. [8] examined the infection route by conducting epidemiologic investigations on super-spreaders and secondary infected patients who infected 82 people in an emergency room at the Samsung Medical Center. It was found that several infected patients were not in direct contact with the super-spreaders, and the infection was not caused by a contaminated environment, given that no patient or medical staff was infected after the isolation of the super-spreaders. Nam et al. [9] and Park et al. [10] conducted epidemiologic investigations on the clinical symptoms and medical environment of other super-spreaders and infected patients who spread secondary infections in the Daejeon area, and identified close contact as the main infection route. The study by Wong et al. [11], who compared the super-spreaders of MERS, Severe Acute Respiratory Syndrome (SARS), and Ebola, revealed that super-spreaders play a significant role in large-scale infection cases. It is important to identify and prevent any possible infection route of such super-spreaders, regardless of its relative probability.

Furthermore, MERS is known to spread mainly by large droplets through close contact, similar to SARS, which is also caused by a coronavirus (CoV) [12]. However, given that close contact does not explain the spread of infection, as suggested by the previously mentioned epidemiologic studies, infections via other routes, such as fomites and air, were suspected [13,14]. In the case of SARS, which spread from Hong Kong and China and resulted in the infection of 8096 patients worldwide in 2003 [15], close contact was identified as one of the infection routes, and the possibility of infection through the air was suggested in several studies [16]. Li et al. [17], Yu et al. [18], and Qian et al. [19] suggested the possibility of infection through the air for secondary infected patients, for whom close contact could not be established as the infection route. In addition, the importance of ventilation in the control of infections indoors was emphasized using epidemiologic investigations and numerical analyses in the Prince of Wales hospital, in which the initial infected patient was known to have infected 125 secondary patients. Previous studies in South Korea were mainly focused on the identification of the infection route via close contact, based on the epidemiologic characteristics of group infections caused by the initial MERS patient. However, it is necessary to examine all possible infection routes with respect to super-spreaders, as with the SARS CoV.

The aim of this study was to identify the airflow as a possible infection routes of secondary infected patients, for whom close contact was not identified, using the spatial distribution and environmental analysis of Pyeongtaek St. Mary’s Hospital, in which the initial MERS patient directly and indirectly infected 38 people after being admitted to the hospital on 15 May 2015, for two nights and three days. Thus, the possibility of the spread of the MERS infection via the indoor airflow was examined by the analysis of the ventilation systems, and the results of an epidemiologic investigation conducted to identify the infection routes in the hospital [20].

## 2. Materials and Methods

### 2.1. Epidemiological Investigation of the Hospital

The hospital, located 65 km south of Seoul, was built at the beginning of 2015, and was only in operation for several months prior to the outbreak. It is a nine-story building with inpatient wards on the fourth to the ninth floors. The initial patient was admitted on the eighth floor for three days, where 38 patients were directly or indirectly infected, including family members and hospital staff on the seventh and eighth floors. The initial patient was assumed to mostly cause infections on the eighth floor, and infections on the seventh floor spread through secondary infected patients after the initial patient had been released the hospital, according to the epidemiological investigation [7].

An extended epidemiological investigation was conducted during June 2015 through interviews with patients, families and medical staff who stayed at the eighth floor to identify the transmission routes during the three days. We also investigated the building systems, windows and doors on the eighth floor because their conditions are closely related with indoor airflow. Tracer experiment was also conducted as an epidemiological investigation to identify the airflow and possible dispersion of infectious aerosols through the airflow.

### 2.2. Tracer Experiments

We conducted tracer experiments by replicating the conditions of the initial two nights and three days after the initial patient was admitted to the hospital. The entire hospital was closed on 29 May 2015, upon the confirmation of the MERS group infection, and a primary disinfection was performed in the hospital. Thereafter, a tracer experiment was conducted on 7 June 2015. In the experiment, the conditions of the hospital during the occurrence of the infection were maintained except patients and medical staffs who were isolated in other facilities.

**Figure 1:**
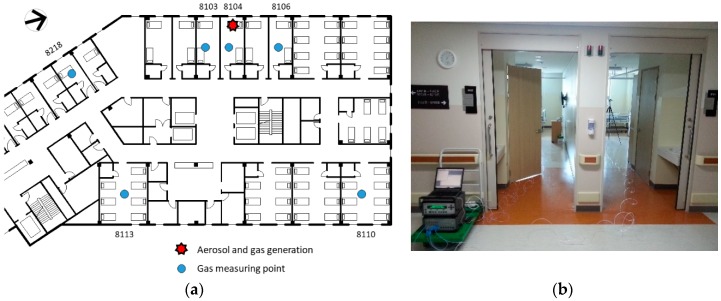
Setup of the tracer experiment using sulfur hexafluoride (SF6) gas: (**a**) dosing and sampling points; (**b**) sliding doors of rooms 8103 and 8104.

A tracer experiment was conducted using sulfur hexafluoride (SF6) gas as the tracer gas. SF6 gas is commonly used to trace the dispersion of pollution substances or measure the ventilation performance, given that it has a low toxicity and seldom exists in normal indoor environments. The tracer gas was continuously generated on the bed in the room of the initial patient, at a rate of 1 L/min. The concentration of SF6 was measured at a height of 1.2 m at the centers of room 8104, rooms 8103 and 8106 (adjacent rooms), rooms 8110 and 8113 (rooms across the corridor), and room 8218 (a single-patient room), using a photoacoustic multi-gas monitor (1412, LumaSense Technologies, Inc., Santa Clara, CA, USA), as shown in Figure 1. At the end of tracer gas experiment, the airflow near room 8104 was visualized and verified using a smoke generator.

During the experiment, the conditions of the eighth floor were maintained, as during the MERS outbreak, with the exception of the external conditions. All the windows were open, and the air conditioning systems were not operated, according to the epidemiological investigation. The operation of the ventilation systems and the opening of the doors were changed during the experiment, as their status could not be confirmed from the investigation. The movement of the experimenter wearing personal protective equipment was minimized throughout the experiment.

## 3. Results

### 3.1. Epidemiological Investigation

**Figure 2:**
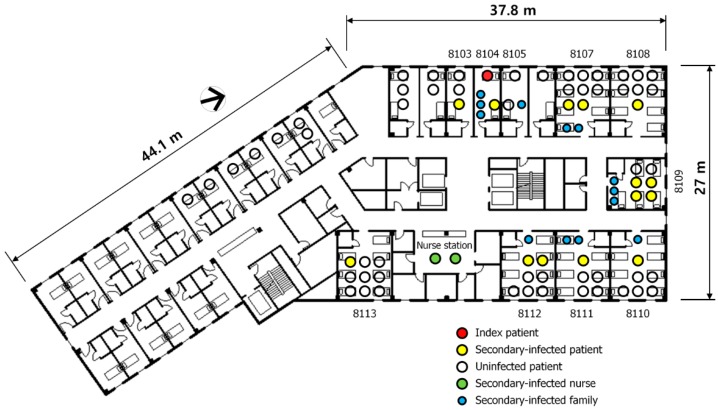
Map showing infection on the eighth floor of Pyeongtaek St. Mary’s Hospital.

From the epidemiological investigation, a patient and a caregiver who remained for an additional half of a day, in addition to two caregivers of the first patient who remained on the window side of the bed in room 8104, were all infected, as shown in Figure 2. In each of the adjacent rooms (8103 and 8105), one patient and one caregiver were infected. In two 7-patient rooms (rooms 8107 and 8108) along the same corridor, three patients and two caregivers were infected. Recently, Song et al. [21] conducted a seroepidemiologic investigation and confirmed that one patient in room 8102 was also infected, without the display of symptoms. In addition to the adjacent rooms, nine patients and seven caregivers were infected in room 8109, which is 15 m from room 8104, in addition to the rooms across the corridor (rooms 8110–8113). This could be explained by the conjecture that the range of the MERS infection, which is known to spread through droplets, is within 2 m. Although contact by the movement of the infected patients was also suspected, there were many secondary infected patients, for whom contact due to the movement of the initial patient was not confirmed. The initial patient did not walk around the rooms of the other patients, and only used the elevator for transport to medical examinations, in addition to walking outside. Close contact with other patients in the elevator or in the hospital was also limited, according to the interview with the patient and the review of the closed-circuit television footage. It was therefore necessary to examine the possible infection routes through fomites or air. Nevertheless, no infected patients were reported in the maternity ward with single-patient rooms, which is located in the south.

There were 19 single-patient rooms, six 2-patient rooms, six 7-patient rooms, and one 5-patient room on the eighth floor. Each patient room is cooled using air conditioners installed on the window side of the ceiling, and heated by hot-water radiators installed on the window side of the floor. Moreover, heat recovery ventilation units were installed for the ventilation of 2–6 rooms. Supply diffusers with diameters of 300 mm were located at the window side, and exhaust vents of the same size were located at the door side. The locations and sizes of all the diffusers and vents were identical in all the patient rooms, and the air change rate of the unit was approximately four air changes per hour (ACH). Each room has an affiliated toilet with exhaust air of 100 m^3^/h, and a small project window for natural ventilation, which can be opened or closed. However, only room 8104, in which the initial patient was hospitalized, does not have a ventilation system. The air conditioner in each room can be operated by the patient, using the operation switch located in the room. In the corridor, air conditioners were installed in the ceiling without any ventilation systems. The air conditioning systems were seldom operated, and the windows were mostly open, due to the mild outdoor temperature, with the exception of night time, according to our epidemiological investigation.

### 3.2. Tracer Experiments

**Figure 3:**
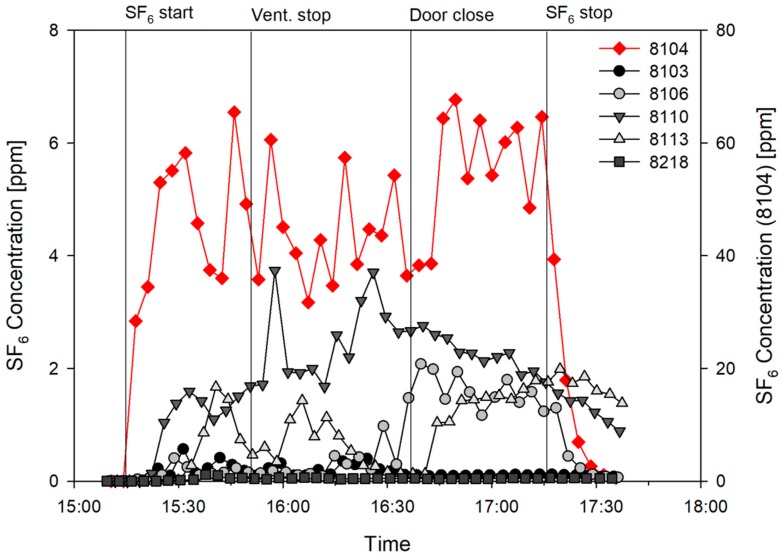
Tracer gas experimental results.

As shown in Figure 3, the concentration in room 8104 reached 53 ppm in approximately 10 min, and the average concentration of 49.5 ppm (standard deviation 10.9 ppm) was maintained until the gas generation was stopped after 2 h. After the door was closed, the average concentration increased from 10 ppm to 60 ppm. All other rooms exhibited a concentration increase according to that of room 8104; however, the degree and time of the increase varied, depending on the room. First, the concentration increased in adjacent rooms 8106 and 8103, and room 8110, approximately 8 min after the gas generation. In particular, the concentration increased significantly to 1.59 ppm (3.2% of the average concentration of room 8104) in room 8110 approximately 18 min after the SF6 gas generation, and maintained the highest concentration (average of 2.09 ppm and maximum of 3.74 ppm) among the rooms, with the exception of room 8104, until the SF6 gas generation was stopped. In contrast, the concentration increased to 0.56 ppm in adjacent room 8103, and decreased after the door was closed, thus reaching an average value of 0.18 ppm. In room 8106, the concentration was relatively low, similar to that of room 8103; however, it increased to 2.07 ppm, and maintained an average value of 1.63 ppm until the SF6 gas generation was stopped. In room 8113, which is opposite room 8104 but farther to the south, the concentration increased approximately 15 min after the SF6 gas generation, which was somewhat delayed when compared with those of the other rooms. Moreover, it reached 1.67 ppm, decreased, and then maintained an average value of 0.63 ppm. After the door was closed, the concentration of room 8104 increased by approximately 20%. However, the concentrations of the other rooms did not decrease until the SF6 gas generation was stopped. The airflow through the undercut of the sliding door, which was also verified by the subsequent smoke test, was assumed to maintain the dispersion of the tracer gas to the other patient rooms. In room 8218, which is a single-patient room in the maternity ward to the south of room 8104 where no MERS patient was admitted, the concentration increased to 0.12 ppm after the initial SF6 gas generation, and maintained the lowest concentration with an average value of 0.048 ppm. After the gas generation was stopped, there was a rapid decrease in the concentration in room 8104, in addition to that in room 8106. However, the concentrations in rooms 8110 and 8113 decreased gradually.

Overall, room 8110, which was the farthest from the source, had SF6 concentrations similar to or higher than those of the rooms adjacent to room 8104. Moreover, the attack rate of room 8110 was also the highest among the measured rooms, as shown in Table 1.

### 3.3. Airflow

**Figure 4:**
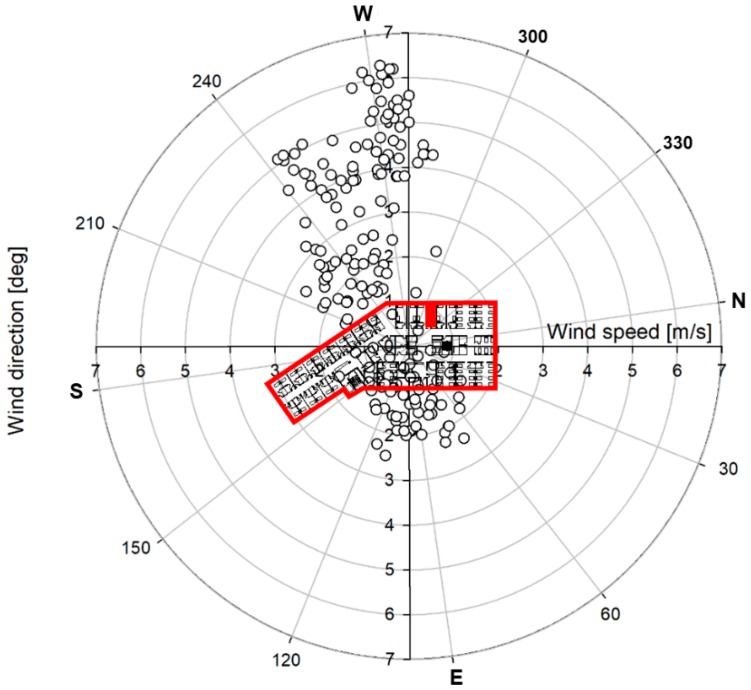
Wind direction and speed measured at a weather station of National Weather Service located 3.6 km away from the hospital during the tracer experiment.

After the tracer gas experiment, the airflow from room 8104 to the corridor was confirmed using a smoke generator. The airflow could be attributed to the presence of strong westerly winds when the tracer-gas experiment and smoke test were conducted, given that wind was introduced to room 8104 through the open window and flowed out toward the corridor, as shown in Figure 4. During the tracer gas experiment, the air change rate estimated using the average concentration of 49.5 ppm and the emission rate of SF6 before closing the door during was approximately 20 ACH. A large amount of outside air was introduced by strong westerly winds through the relatively small window of the room at approximately 17 ACH, even after the door was closed. The air change rate estimated by stopping the generation of the tracer gas and using the attenuating concentration was also high, at approximately 15 ACH. When the air change rate is high, the concentration of pollutants can be diluted and dispersed indoors through the corridor if they are not discharged outside through ventilation ducts. Therefore, the causes of the dispersion of the tracer gas from room 8104 to the other rooms through the corridor, along with the introduced wind, could be estimated using the results of the tracer-gas experiment. In addition, the gas from room 8104 did not disperse to room 8218, given that the outdoor airflow in the west–southwest direction was introduced to the maternity ward through the windows.

## 4. Discussion

The results of the tracer-gas experiment revealed that the highest concentration of SF6 gas from room 8104 was in room 8110, which is one of the rooms on the east side. Although MERS is known to spread mainly by droplets via close contact as mentioned earlier, several infection cases could not be explained by droplet infection. In a study by Xiao et al. [13], the multi-route analysis of the MERS outbreak was conducted in the same hospital. The conditions in the hospital during the three-day period were assumed, and the probabilities of the airborne, close contact, and fomite infection routes were analyzed and compared. However, there was a low probability of close contact with the initial patient, according to the epidemiological investigation and interviews with the infected patients in the patient rooms far away from room 8104. Moreover, the average attack rate of the rooms on the west side, adjacent to room 8104, was 24.1% and that of the rooms on the east side, far away from room 8104, was 40.0%. Therefore, in this study, the possibility of long-distance infection through the air was not assumed to be low, and was examined using the tracer-gas experiments.

According to the Centers for Disease Control and Prevention (CDC) [22], airborne precautions should be taken with respect to MERS-infected patients. This is because droplets containing viable MERS-CoV could be transferred over large distances in certain environments such as healthcare facilities, where large amounts of droplets could be released during coughing or medical treatments, and transferred over distances significantly larger than 1–2 m by airflow. The propagation distance and time of droplets or droplet nuclei in the air may increase, depending on the indoor environment [23,24], which may be affected by mechanical or natural ventilation. Bin et al. [25] and Kim et al. [26] performed the environmental sampling of negative-pressure isolation rooms, in which MERS-confirmed patients were hospitalized. Moreover, MERS-CoV was detected in the ventilation ducts in the ceiling. The detection of MERS-CoV in the air-conditioner filter in the ceiling of the room opposite room 8104, among the unpublished environmental sampling results of the hospital, also indicates that the droplets or droplet nuclei from patients can travel over large distances in certain environments. Though investigations were conducted, the operation status of the ventilation system and air conditioner installed in the room of the hospital during the stay of the initial patient could not be clearly determined. The control devices of the ventilation system and air-conditioner were in the room, and the patient had access to them. However, the cooling or heating system was not operated, given that the average temperature of Pyeongtaek was 17.8 °C (10.3–26.4 °C) from 15 to 17 May when the initial patient was hospitalized, according to the epidemiological investigation. Moreover, it was not clear whether the ventilation system was operated during the outbreak. Furthermore, room 8104 did not have a ventilation system, due to a construction flaw. Apart from the ventilation system, each room had a small project window for natural ventilation, which could be opened or closed by the occupants of the room. The window was opened for ventilation, given that the outside air was mild, with the exception of the night time. It is necessary to examine the effects of the introduction of the outside air through the window, and the effect of the indoor airflow formed by the outside air on the dispersion of droplets or droplet nuclei in room 8104.

**Figure 5:**
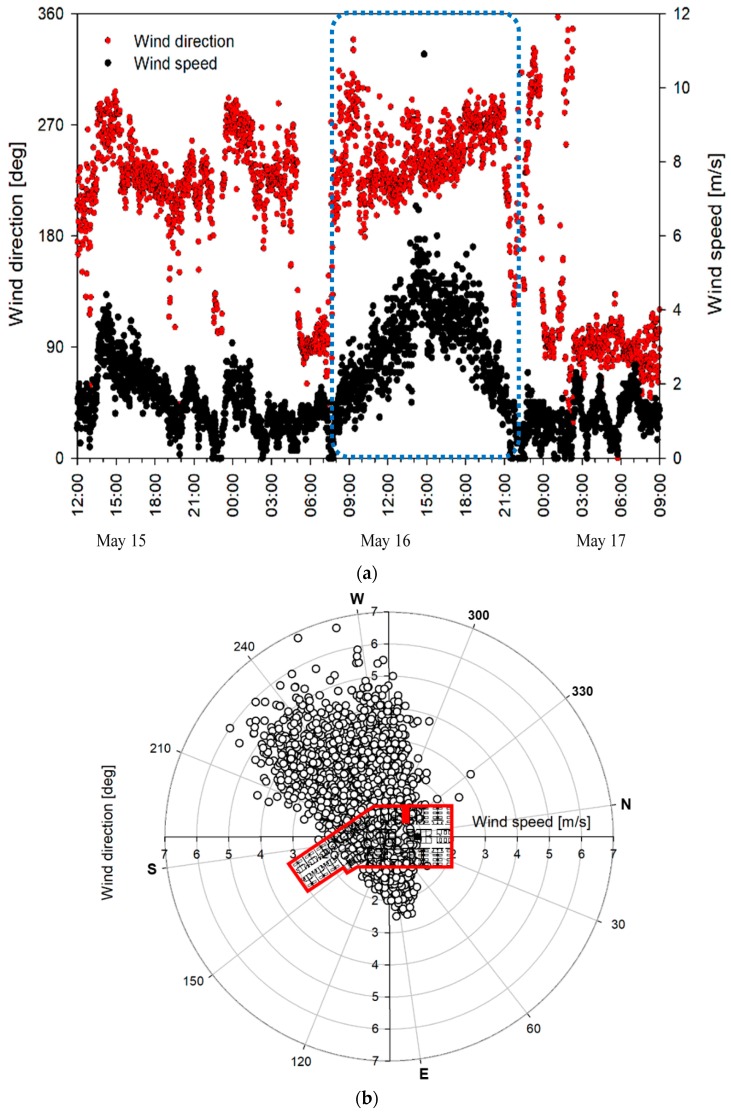
Outdoor airflow during the stay of the initial patient: (**a**) wind profile; (**b**) wind rose graph.

Figure 5 presents the wind direction and wind-speed distribution measured at the same weather station with Figure 4. From 15 to 17 May when the initial patient was hospitalized, the main wind was that in the west–southwest direction, and the wind speed in this direction was high. In particular, on May 16, the average wind speed was 2.15 m/s, the maximum wind speed was 10.9 m/s, and the wind was mostly in the west–southwest wind direction. Therefore, it is possible that airborne particles from room 8104 were introduced to the corridor by the outside air entering through the window when the window and room door were open. This is similar to the external airflow observed when the tracer experiment was conducted using SF6 gas. The tracer experiment also confirmed that pollutants from room 8014 were dispersed into adjacent rooms and rooms on the opposite side. Some of the tracer gas was assumed to flow into adjacent rooms 8103 and 8106, in addition to the dominant dispersal into the patient rooms on the opposite side, due to changes in the wind direction and speed, and the inflow of exhaust air to the affiliated toilets of adjacent rooms during the experiment. Previous experiments and the results of the computational fluid dynamics (CFD) analysis conducted in SARS cases also confirmed the possibility of long-distance dispersion due to indoor airflow [17,18,19,27]. In addition, it is also possible that a high concentration of airborne particles, including the MERS virus, accumulated in room 8104, which does not have a ventilation system, given that its window and door were closed at night. Thereafter, the particles may have been discharged in large amounts by the opening of the window and door in the morning.

According to the World Health Organization (WHO) [28], with respect to the layout of the patient rooms and corridors for infection management, a single-side corridor, courtyard, or atrium type may be used for natural ventilation. However, the central corridor type is not appropriate, given that infectious viruses from one room may travel to opposite rooms through the corridor due to the natural ventilation. Pyeongtaek St. Mary’s Hospital has a central corridor layout; thus, it is highly probable that viruses spread into opposite rooms through the corridor as shown in the results of tracer experiment.

In this study, the dispersion of MERS-CoV was simulated using tracer gas. This experiment has been widely used to observe the dispersion phenomenon of airborne bacteria [29,30]. However, at sites different to controlled laboratories, it is difficult to derive reproducible results due to the interference from high-concentration airborne particles already present in indoor and outside air. Although the SF6 gas used in the tracer-gas experiment is seldom detected in normal environments, and therefore not subject to interference, it exhibits an aerodynamic dispersion behavior different from that of particles, as it contains gas molecules smaller than particles. In previous studies on SARS [17,27], airflow indoors and outdoors was analyzed using passive contaminant to identify the dispersion of SARS virus based on the assumption that the droplets exhaled from the infected patient are evaporated very quickly and get smaller enough to flow like airflow. For the same reason, SF6 gas is widely used for the dispersion of pollution substances such as gas and airborne particles, or for airflow examinations, as it can be used to simulate airflow [31,32]. Besides, we tried to conduct the tracer experiment in the same condition with the initial outbreak period at the hospital. However, the patients, medical staff and their movements could not be simulated because the hospital was evacuated and they were isolated in other facilities. Moreover, the hospital was still under risk of contamination. The movements and heat generation from the occupants could accelerate the dispersion of air and particles from the initial patient room.

The tracer gas was not measured in several important patient rooms such as 8108, 8109 and 8111 due to the limited supply of measuring equipment. Moreover, the effect of the heat generation of the occupants in each room was not simulated. The experiment was prepared and conducted on 8 June 2015, approximately 1 week after the hospital was evacuated, to determine the infection route (including the airborne route), which was not confirmed at that time. The measuring equipment was therefore limited due to the urgent demand. A more precise analysis for all the patient rooms may be conducted in future work, using CFD simulations.

We identified the dispersion of tracer gas from the initial patient room to long-distance patient rooms, which implies that airflow could be an infection route. The results also correspond epidemiologically with attack rates. However, the correspondence does not directly indicate infection by the dispersed portion of tracer gas from the initial patient room, because biological factors such as host susceptibility and infective dose of MERS-CoV are not yet clear.

## 5. Conclusions

In this study, epidemiological investigation and tracer experiments were conducted to identify airflow as a possible infection route. This was achieved with the exception of the droplet infection route identified in Pyeongtaek St. Mary’s Hospital. The conclusions of the study can be summarized as follows:

1. The tracer gas from room 8104, in which the initial patient (super-spreader) was hospitalized, was confirmed to spread over long distances to patient rooms across the corridor. This indicates the significant effect of the outdoor wind entering through the window.
2. The high concentration in room 8104 was probably spread to the corridor and rooms on the opposite side due to the strong airflow entering from the outside.
3. The results indicate that cross ventilation by outdoor wind in central corridor inpatient ward could cause dispersion of infectious aerosols to indoor through airflow.
4. Although there were limitations in confirming the infectivity of propagated airborne particles, the possibility of the spread of infections by airflow was presented for the analysis of relatively long-distance infection cases, for which the close-contact infection route by droplets could not be identified.

Precautions are essential for every mode of unknown or known infectious diseases, especially in their early stages of outbreak. For example, the MERS-confirmed and MERS-suspected patients were quarantined in the negative pressure isolation wards that were primarily intended for airborne infection patients. The findings in this study can be used in initial epidemiological investigations and precautions, with respect to outbreaks of MERS or similar unknown infectious diseases.

## Figures and Tables

**Table 1:** Average concentration of SF6 in patient rooms (ppm). The values in parentheses indicate the ratios of the SF6 concentration to the average concentration in room 8104.

Room Number	8104	8103	8106	8110	8113	8218
Average	49.5	0.18	0.71	2.09	0.63	0.048
	(100%)	(0.36%)	(1.43%)	(4.22%)	(1.27%)	(0.10%)
Maximum	67.7	0.56	2.07	3.74	1.67	0.12
	(137%)	(1.13%)	(4.18%)	(7.56%)	(3.37%)	(0.24%)
Infected case	5/5	1/3	0/1	2/6	1/9	0/1
Distance from 8104	0 m	1 m	6.3 m	28 m	24 m	18 m
